# Supplementary figures and images for: Generation of a genetically double-attenuated Plasmodium berghei parasite that fully arrests growth during late liver stage development
Source: PLoS One. 2024 Dec 31;19(12):e0316164. doi: 10.1371/journal.pone.0316164 (PMC11687666; doi:10.1371/journal.pone.0316164)

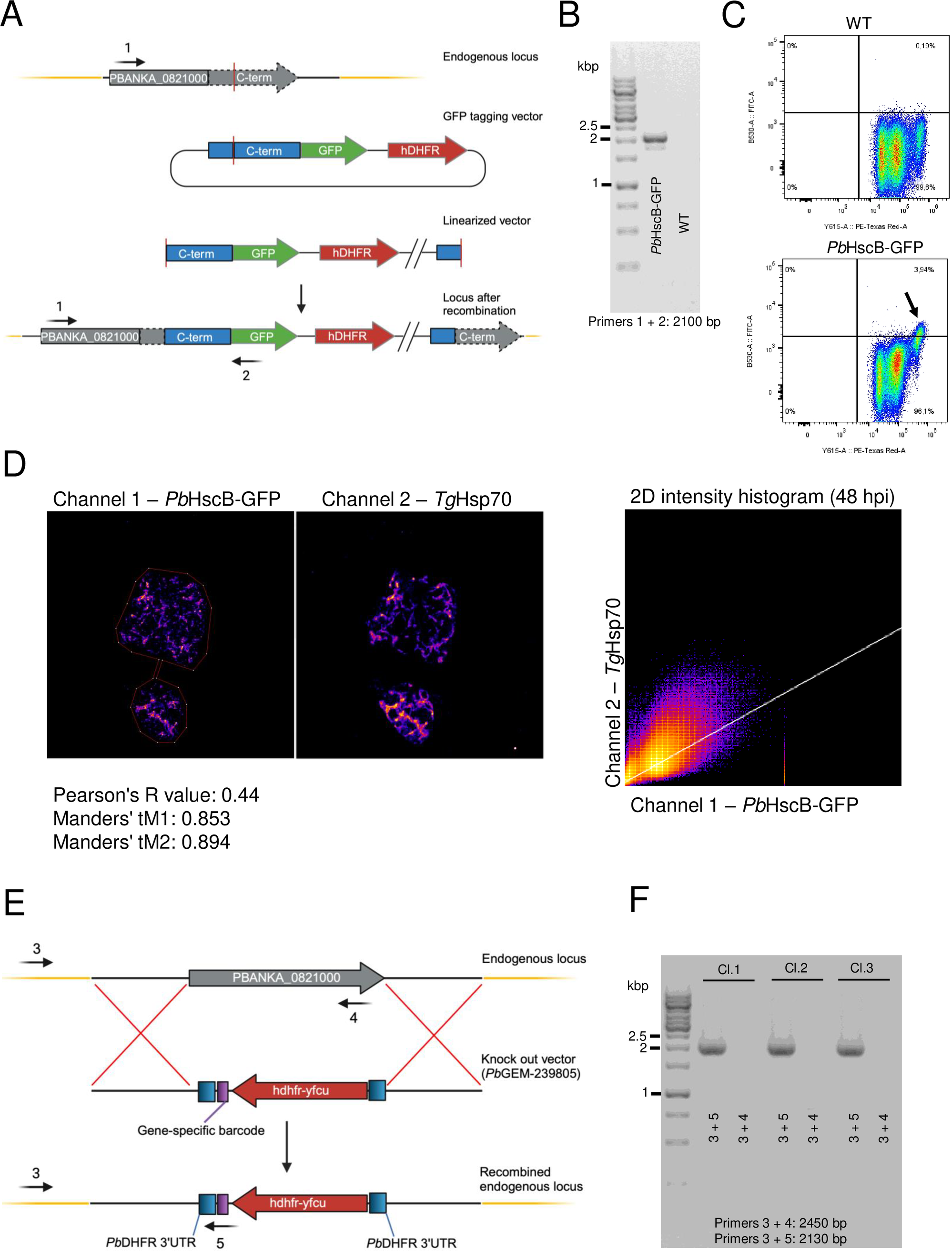

Supplement: S1 Fig — (A) Schematic representation of single crossover recombination of GFP-tagging vector at the PBANKA_0821000 locus. The C-terminus of PBANKA_0821000 (blue) was amplified and fused to a C-terminal GFP. Human dihydrofolate reductase (hdhfr) was used as a positive selection marker. Primers used to check for integration are marked as 1 and 2. (B) Agarose gel showing products of PCR done on genomic DNA extracted from WT and from transgenic PbHscB-GFP parasites. Expected band size for the PCR: 2,100 bp. (C) Flow cytometry graph from WT and PbHscB-GFP parasites showing mCherry-expressing parasites with a population of GFP-expressing parasites (green arrow). (D) Represents the Channel 1 (PbHscB-GFP) and the Channel 2 (TgHsp70) at 48 hpi timepoint (left panel) used to measure the Pearson correlation coefficient (PCC) and the Mander’s overlap coefficient (MOC). The 2D intensity histogram of Channel 1 and Channel 2 (right panel) shows the ratio of intensities at the exact position in a density heat map (a high intensity is displayed in yellow, a low intensity in purple). The image at 48 hpi used correspond to the one presented in Fig 1B. (E) Schematic representation of double crossover recombination of the PbHscB-KO vector (PbGEM-239805) at the PBANKA_0821000 locus. Human dihydrofolate reductase (hdhfr) was used as a positive selection marker and yeast cytosine deaminase uracil phosphoribosyl transferase (yfcu) as a negative selection marker. The purple bar represents the gene-specific barcode. Blue bars represent PbDHFR 3’ UTRs. Primers used to check for integration are marked as 3–5. (F) Agarose gel showing PCR products done on genomic DNA of PbHscB-KO parasites after limiting dilution. Expected band sizes are 2,450 bp for 3+4 (WT) and 2,130 bp for 3+5 (locus after integration of KO vector). Clone 1 was chosen for further analysis. Schematics created with BioRender.com. (TIF) [file pone.0316164.s001.tif]

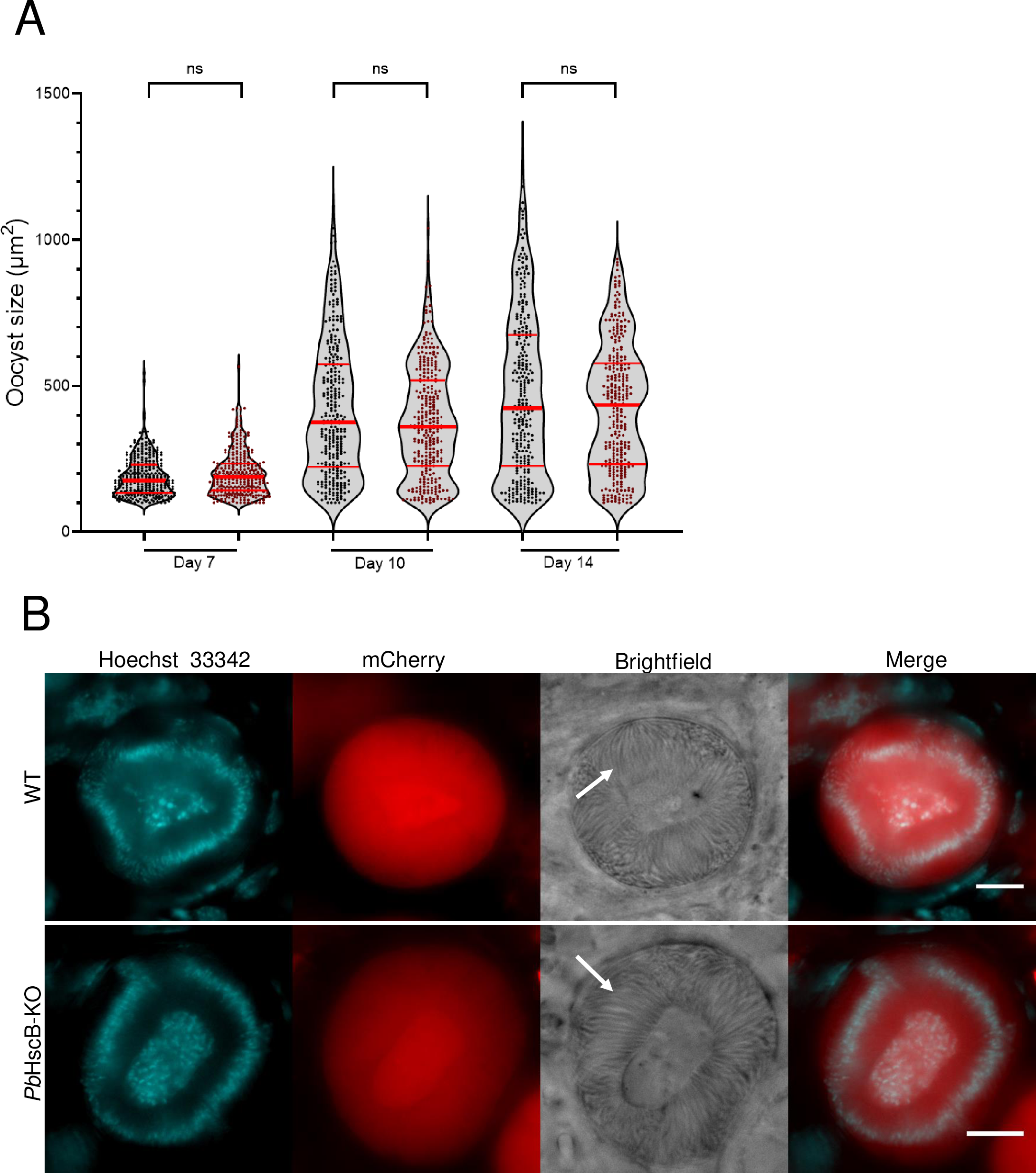

Supplement: S2 Fig — (A) Midguts of infected mosquitoes (n = 10, black: WT and red: PbHscB-KO) were dissected and imaged on day 7, day 10, and day 14 post-feed. Images were acquired using a 5x objective and analyzed using ImageJ software: mCherry signal was binarized using the threshold function and the oocyst size was measured with the ‘analyze particle’s function. The shown result is a representative of three experiments and shows the size of 300 oocysts per parasite line as violin plot with individual oocyst sizes and medians with interquartile range. Kruskal-Wallis test did not result in a significant difference (ns) between WT and KO oocysts in none of the analyzed time points. (pday7 = 0.9412, pday10 = 0.4174, pday14>0.9999). (B) Infected midguts were extracted on day 14 post-feed and DNA was stained with Hoechst 33342 (cyan). Cytosolic mCherry is shown in red. Sporozoite formation in PbHscB-KO oocysts is highlighted with a white arrow. Scale bars are 10 μm. (TIF) [file pone.0316164.s002.tif]

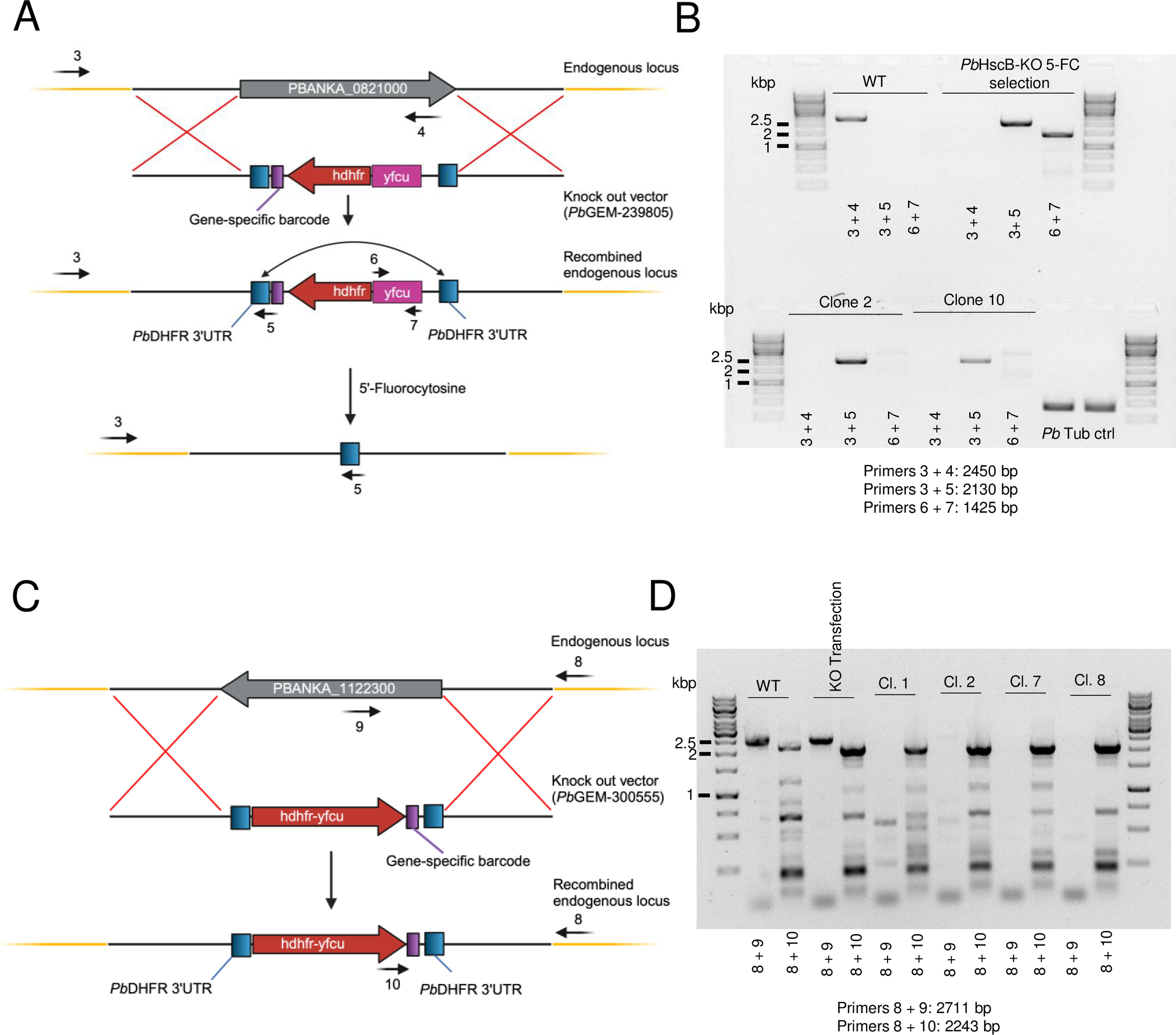

Supplement: S3 Fig — (A) Schematic representation of negative selection using 5-fluorocytosine (5-FC). PbDHFR-3’UTRs can recombine, which removes the selection cassette. Marker-free parasites can be selected using 5-FC. The absence of the selection marker was checked with indicated primers 3–7. (B) Agarose gel showing PCR products done on genomic DNA of Pb1868 WT parasites, 5-FC selected parasites, and two 5-FC selected clones after limiting dilution. Expected PCR products are 2,450 bp for 3+4; 2,130 bp for 3+5; and 1,425 bp for 6+7. The absence of a selection marker was confirmed for clone 2, which was selected for the generation of the double KO line. (C) Schematic representation of double crossover recombination of the PbMei2-KO vector (PbGEM-300555) at the PBANKA_1122300 locus. Human dihydrofolate reductase (hdhfr) was used as a positive selection marker and yeast cytosine deaminase uracil phosphoribosyl transferase (yfcu) as a negative selection marker. The purple bar represents the gene-specific barcode. Blue bars represent PbDHFR 3’ UTRs. Primers used to check for integration are marked as 8–10. (D) Agarose gel showing PCR products done on genomic DNA of Pb1868 WT and PbHscB-PbMei2-dKO parasites after transfection and after limiting dilution. Expected band sizes are 2,711 bp for 8+10 (WT) and 2,243 bp for 8+9 (locus after integration of KO vector). Clone 1 was chosen for further analysis. Schematics created with BioRender.com. (TIF) [file pone.0316164.s003.tif]
