# Supplementary material for: Generation of a genetically double-attenuated Plasmodium berghei parasite that fully arrests growth during late liver stage development
Source: PLoS One. 2024 Dec 31;19(12):e0316164. doi: 10.1371/journal.pone.0316164 (PMC11687666; doi:10.1371/journal.pone.0316164)

Raw images of agarose gels where PCR products were loaded on

From Supplementary Figure 1

Supplementary Figure 1B

Integration PCR of the *Pb*HscB-GFP vector

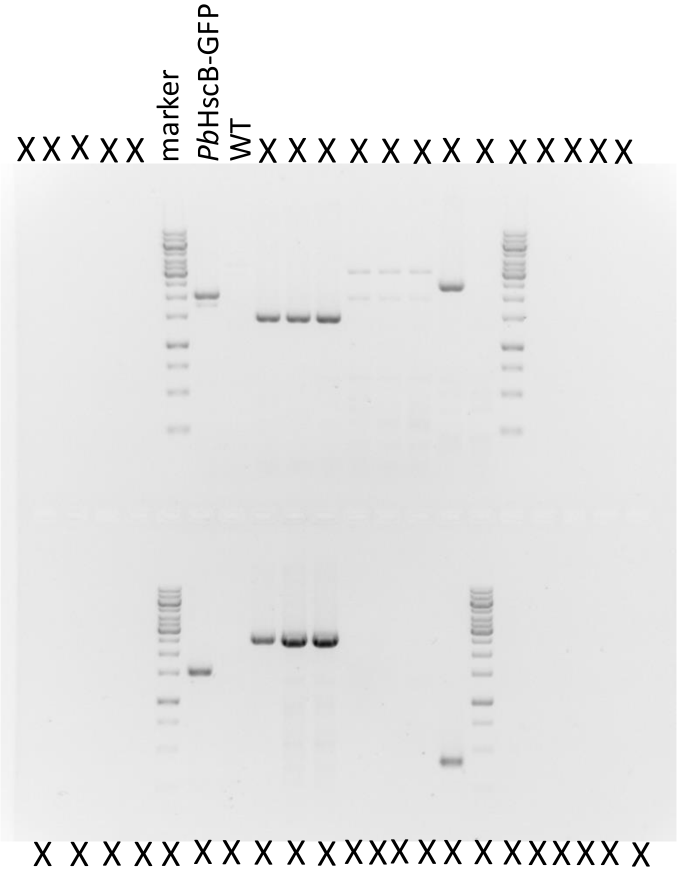

Supplementary Figure 1F

Integration PCR of the *Pb*HscB-KO vector

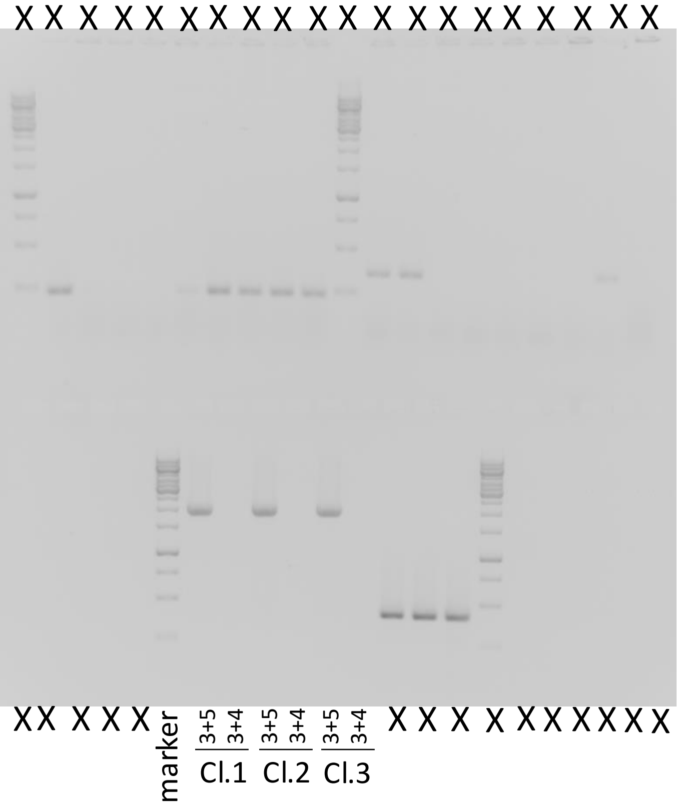

Raw images of agarose gels where PCR products were loaded on

From Supplementary Figure 3

Supplementary Figure 3B

Creation of the *Pb*HscB-KO-marker free parasite line

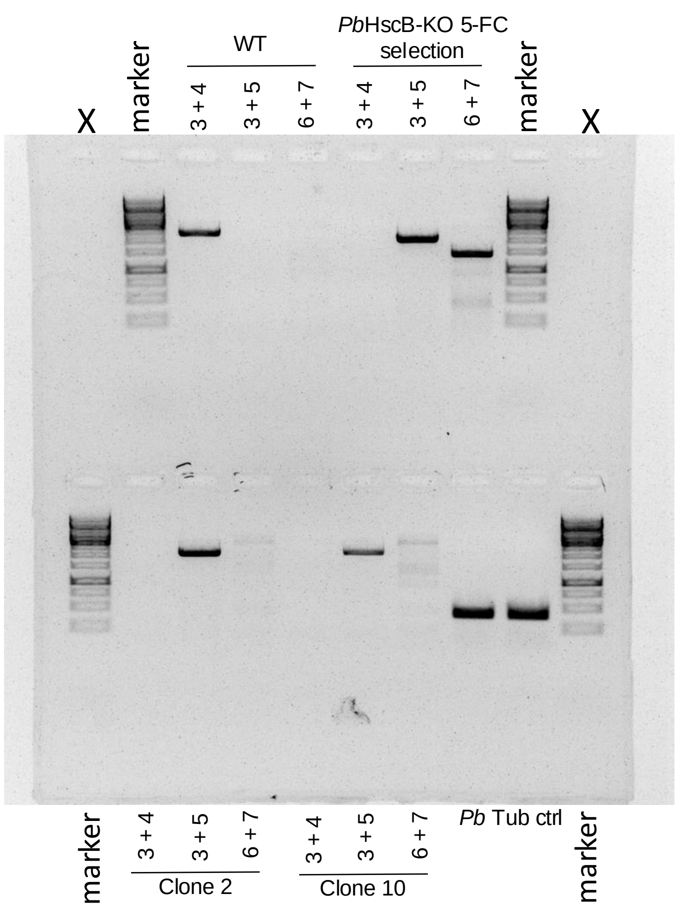

Supplementary Figure 3D

Integration PCR of the *Pb*HscB-*Pb*Mei2-dKO vector

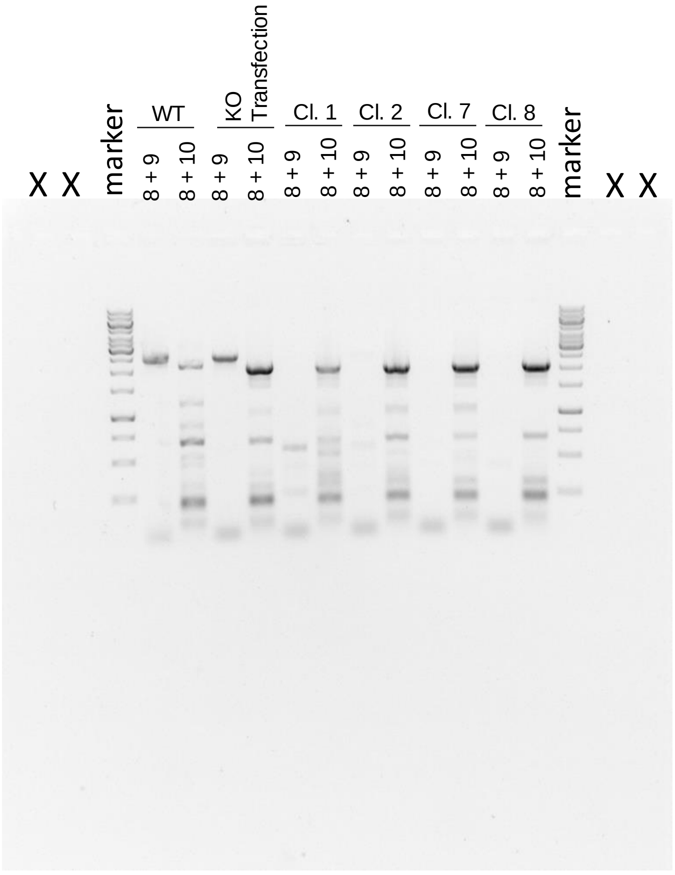

Supplement: S1 Raw image — (PDF) [file pone.0316164.s004.pdf]
