# Supplementary material for: Generation of a genetically double-attenuated Plasmodium berghei parasite that fully arrests growth during late liver stage development
Source: PLoS One. 2024 Dec 31;19(12):e0316164. doi: 10.1371/journal.pone.0316164 (PMC11687666; doi:10.1371/journal.pone.0316164)
Supplement: Attachment — Submitted filename: Response to Reviewers.docx [file pone.0316164.s005.docx]

**Responses to the Reviewers:**

We strongly appreciate the Reviewers’s comments to improve and clarify our work. We made the changes accordingly and responded to all the requests in blue below the comments.

**REVIEWER COMMENTS:**

**Reviewer #1:** This is a nice and straight forward paper describing the generation of a new double gene deletion mutant in Plasmodium berghei as a model for attenuated parasites. Clearly further tests are needed as the authors state in the discussion but as a first report the paper is technically sound, provides a new and interesting tool and novel insights. The following are suggestions for slight improvement:

What is not clear to me is if MSP1 is expressed in mei2/linup KOs. This could be interesting and a possible point to discuss: If no MSP1 is expressed likely it’s not very late-stage development and no antibodies are made to protect from a blood stage infection. Hence, a breakthrough infection will likely lead to full blown infection.

Thank you for your insightful comment. Indeed, in our study, there was no detectable MSP1 expression in the *Pb*HscB-*Pb*Mei2-double knockout (dKO) parasites. This observation aligns with the expectation of limited cross-reaction and suggests that the parasites do not reach very late-stage liver development. The absence of MSP1 expression is significant because it implies that antibodies targeting this protein, which could contribute to protection against blood-stage infection, are not generated.

This raises an interesting point for discussion: if MSP1 is not expressed in double knockouts, the parasites may not progress to the stage where protective antigens are sufficiently presented to elicit an immune response. Consequently, should a breakthrough infection occur, it could likely result in a complete blood-stage infection due to the absence of prior protective immunity. We will highlight this consideration in the revised discussion to emphasize the implications of MSP1 absence and its potential impact on immune protection and breakthrough infections.

Lines 41/42: new data from Sinnis Lab suggests that Anopheles transmits 500-1000 SPZ, please modify (PMID: 38272943)

Reference added and changes made.

43: also transmigrate through skin cells (PMID: 18312843)

Reference added and changes made.

56: new data suggest under 20% efficacy in implementation studies

Change made.

94/95 This paper PMID: 27241521 reports the lowest rate of breakthrough in a GAP to my knowledge and should be mentioned if low number of mice are used to test for breakthroughs – could also be discussed in the paragraph around line 460

We appreciate the reviewer’s suggestion regarding the inclusion of the study reported in Kumar et al, 2016. This paper will be cited to highlight an example of a GAP model with a low breakthrough rate, which is relevant to our discussion on the potential limitations of using a small number of mice for breakthrough testing. We will incorporate this reference in our revised manuscript to provide context and comparison with our findings.

482: please change ‘prove’ into ‘test’

Change made.

**Reviewer #2:** Among the different vaccine approaches against malaria, immunization with whole attenuated sporozoites is considered an interesting strategy since it allows to induce sterilizing immunity based on immune mechanisms directed against the hepatic stages of malaria, the initial phase of parasite multiplication in the vertebrate host.
While the first vaccination tests dating back to the 1970s used sporozoites attenuated by irradiation, current strategies focus on using either non-attenuated parasites with drug cover (« chemically attenuated »), or developping genetically attenuated parasites. This latter solution, requiring less medical monitoring constraints, appears most promising provided the attenuation is robust enough to prevent any breakthrough infections when immunizing with live sporozoites.

In their manuscript entitled " Generation of a genetically double-attenuated Plasmodium berghei parasite that fully arrests growth during late liver stage development" Schmid et al. first investigate the localization of HscB in the rodent parasite Plasmodium berghei within mosquitoes and at the liver stage, using a P. berghei parasite that they genetically engineered to express the GFP reporter gene. They documented the localization of PbHscB at the mitochondrial level in oocysts within mosquitoes, and in exo erythrocytic forms in culture.
The authors demonstrated that deleting PbHscB has no effect on the mosquito stage, thus allowing sporozoite production. However, PbHscB knockout parasites exhibit significant growth and maturation defects at the liver stage, resulting in a prolonged patency period when injected into mice, though infection eventually occurs.

The authors then engineered a second parasite with double deletion for both the HscB gene and Pbmei2, previously shown to be essential for the completion of Plasmodium liver stages, although with a significant risk of breakthrough. This double knockout parasite develops normally in mosquitoes, producing infectious sporozoites, but is completely arrested at the liver stage and cannot initiate a blood-stage infection, even when a massive dose of sporozoites is injected into mice.

The manuscript is technically sound with data that generally support the conclusions, including appropriate statistical analysis and biological replicates. All data underlying the conclusions are available in the main and supplementary figures.

However, a few minor points could benefit from additional clarification or analysis:

The authors refer to a phenotypic screening in reference 34 for their choice to study PbHscB in detail. It is unclear where PbHscB is specifically discussed in that reference. The authors should verify this citation.

Thank you for pointing out the need to verify the citation. The reference to Stanway et al., 2019 (now cited as reference 37, PMID: 31730853) is indeed accurate. This publication by our group and collaborators details the identification of key genes involved in *Plasmodium berghei* liver stage development through a high-throughput phenotypic screen. Although the main focus was on metabolic pathways such as fatty acid and amino sugar biosynthesis, the barcode abundance data for structural gene deletions and others like the *Pb*HscB-knockout (PBANKA_0821000) is included in Table S2 of the study by Stanway et al.

Specifically, the *Pb*HscB-KO parasite showed a 19-fold reduction (Log2FC = -4.23) in barcode abundance at the salivary gland sporozoite (SG) to blood-stage transition (B2), indicating significant impairment during liver stage development. This marked reduction drew our attention to HscB as a candidate for further investigation. We have now clarified this in the manuscript.

Please find below the part of table S2 in Stanway et al recapitulating on the HscB KO barcoding throughout the entire life cycle of *P. berghei*:

|  | **B1-MG data, normalized** | | | **MG-SG data, normalized** | | | **SG-B2 data, normalized, BS fitness-corrected** | | | **Blood stage fitness data (Bushell et al., 2017)** | |
| --- | --- | --- | --- | --- | --- | --- | --- | --- | --- | --- | --- |
|  | **Log2-FC** | **SD** | **Power** | **Log2-FC** | **SD** | **Power** | **Log2-FC** | **SD** | **Power** | **Relative growth** | **Phenotype** |
| PBANKA_0821000 | -1.42 | 0.24 | no power | -1.12 | 0.33 | no power | **-4.23** | **0.60** | **reduced** | 0.82 | Slow |

In Figure 1, the authors use mitochondrial markers to conclude colocalization of PbHscB at the mitochondrial level based on confocal microscopy. Merged images alone are often insufficient for confirming colocalization. Utilizing image analysis tools that measure pixel intensity or calculate correlation coefficients, such as the Pearson coefficient, would strengthen this conclusion.

We appreciate the reviewer’s insightful comment regarding the need for quantitative confirmation of colocalization. In response, we have now included additional analysis in Supplementary Figure 1D, which features the Pearson’s correlation coefficient and Manders' overlap coefficients for *P. berghei* liver stage parasites at 48 hours post-infection, based on the image in Figure 1B. The analysis includes channel 1 (*Pb*HscB-GFP) and channel 2 (*Tg*Hsp70) on the left, alongside a 2D intensity histogram of both channels on the right.

The measured Pearson’s R value was 0.44, with Manders' coefficients tM1 at 0.853 and tM2 at 0.894, indicating a moderate level of overlap between the two signals. We have also updated the figure panel numbers in the main text, revised the legend for Supplementary Figure 1, and included details of these calculations in the Materials and Methods section.

These data are now provided in the revised Supplementary Figure 1, complete with an updated figure legend (see below).

**S1 Fig: Generation of transgenic parasite lines. (A)** Schematic representation of single crossover recombination of GFP-tagging vector at the PBANKA_0821000 locus. The C-terminus of PBANKA_0821000 (blue) was amplified and fused to a C-terminal GFP. Human dihydrofolate reductase (hdhfr) was used as a positive selection marker. Primers used to check for integration are marked as 1 and 2. **(B)** Agarose gel showing products of PCR done on genomic DNA extracted from WT and from transgenic *Pb*HscB-GFP parasites. Expected band size for the PCR: 2,100 bp. **(C)** Flow cytometry graph from WT and *Pb*HscB-GFP parasites showing mCherry-expressing parasites with a population of GFP-expressing parasites (green arrow). **(D)** Represents the Channel 1 (*Pb*HscB-GFP) and the Channel 2 (*Tg*Hsp70) at 48 hpi timepoint (left panel) used to measure the Pearson correlation coefficient (PCC) and the Mander’s overlap coefficient (MOC). The 2D intensity histogram of Channel 1 and Channel 2 (right panel) shows the ratio of intensities at the exact position in a density heat map (a high intensity is displayed in yellow, a low intensity in purple). The image at 48 hpi used correspond to the one presented in Fig 1B. **(E)** Schematic representation of double crossover recombination of the *Pb*HscB-KO vector (*Pb*GEM-239805) at the PBANKA_0821000 locus. Human dihydrofolate reductase (hdhfr) was used as a positive selection marker and yeast cytosine deaminase uracil phosphoribosyl transferase (yfcu) as a negative selection marker. The purple bar represents the gene-specific barcode. Blue bars represent *Pb*DHFR 3’ UTRs. Primers used to check for integration are marked as 3 – 5. **(F)** Agarose gel showing PCR products done on genomic DNA of *Pb*HscB-KO parasites after limiting dilution. Expected band sizes are 2,450 bp for 3+4 (WT) and 2,130 bp for 3+5 (locus after integration of KO vector). Clone 1 was chosen for further analysis. Schematics created with BioRender.com.

In Figure 1B, Hela cells are used to study liver stage features of P. berghei, but these cells differ from natural host cells. Including images of PbHscB liver stages in murine hepatocytes or liver slices from infected mice, or at least in an hepatic origin cell line, would provide more relevant data.

We appreciate the Reviewer’s concerns regarding the use of HeLa cells for studying liver stage features of *P. berghei*. It is important to note that the suitability of HeLa cells for *P. berghei* liver stage studies has been well-documented in the literature. Multiple studies have demonstrated that HeLa cells are permissive for *P. berghei* infection and serve as a reliable model for initial *in vitro* assessments of parasite development, particularly for high-throughput or mechanistic investigations. In addition to the *in vitro* data, we have conducted comprehensive *in vivo* experiments in murine models, which demonstrate solid attenuation of the double knockout parasite as attested in Figure 4. We have now verified the *Pb*HscB-*Pb*Mei2-dKO salivary glands sporozoites’ ability to reach and invade mouse hepatocytes *in vivo* and added the new data in Figure 4E. For this, we infected B6 Albino mice with 100,000 WT (positive control) or *Pb*HscB-*Pb*Mei2-dKO salivary glands sporozoites and performed a full-body bioluminescence analysis at 44 hours post-sporozoite infection. The mock (non-infected mosquito salivary glands) was used as a negative control and the number of mosquitoes dissected was the same as for WT. Although the luminescence signal from the double-attenuated parasites is understandably low due to a tenfold size reduction compared to the control (as shown in Figure 4A), we clearly demonstrate that *Pb*HscB-*Pb*Mei2-dKO parasites can reach and invade mouse hepatocytes *in vivo*. This new data has been incorporated into Figure 4E, the figure legend, main text, and the Materials and Methods section:

**Fig 4: *Pb*Mei2-KO and *Pb*HscB-*Pb*Mei2-dKO parasites are arrested in the liver stage. (A)** HeLa cells were infected with either WT or *Pb*HscB-KO sporozoites and imaged at indicated time points using automated microscopy. The shown result is a representative of three experiments and shows parasite size of 500 parasites measured based on cytosolic mCherry signal expressed. The significance of the results was determined with Kruskal-Wallis test; (****: p<0.0001, ns: p_24h1868-Mei2KO_=0.9528, p_48h1868-Mei2KO_=0.076, p_56h1868-Mei2KO_=0.0674). **(B)** The number of parasites was determined at 6 hpi and 48 hpi by automated microscopy and the number at 6 hpi was considered as 100 %. Significance was calculated by Kruskal-Wallis test, with Dunn’s multiple comparison test (*: p=0.0276; ns: p_1868-Mei2KO_>0.9999, p_1868-dKO_=0.4231). Each dot represents an infection experiment. **(C)** HeLa cells infected with WT or *Pb*Mei2-KO parasites were fixed at 56 hpi and stained with DAPI (DNA, blue), anti-*Tg*Hsp70 antibodies (mitochondrion, green), and anti-MSP1 antibodies (parasite plasma membrane, magenta). Cytosolic mCherry signal is shown in red. Scale bars are 10 µm. **(D)** HeLa cells infected with WT or *Pb*Mei2-KO parasites were fixed at 56 hpi and stained with DAPI (DNA, blue) and antibodies against UIS4 (PVM, magenta). Cytosolic mCherry is shown in red. Scale bars are 10 µm. **(E)** Albino mice (B6(C)/Rj-Tyrc/c) injected with 100,000 sporozoites from mosquito salivary glands infected with either *Pb*HscB-*Pb*Mei2-dKO or WT parasites were assessed for luciferase activity via luminescence measurement. Salivary glands from uninfected *P. berghei* mosquitoes served as a negative control (Mock). At 44 hpi mice were anaesthetized, injected with luciferin and parasite load in the liver was analyzed using an In Vivo Imaging System (IVIS). The graph displays the mean total photon counts per second, with each dot representing an individual mouse (N=3 per group). Statistical significance was determined using an unpaired t-test. **(F)** C56BL/6 mice were infected with 100,000 sporozoites each (4 mice for WT, 3 mice for *Pb*HscB-KO, 4 mice for *Pb*Mei2-KO and 6 mice for *Pb*HscB-*Pb*Mei2-dKO). Parasitemia was monitored daily by flow cytometry and a parasitemia of 0.01% (100 parasites per 1 million erythrocytes) was set as threshold. In a pilot study, it was already confirmed that infection of mice with 100,000 *Pb*Mei2-KO sporozoites resulted in breakthrough infections.

Figure 4B assesses the sporozoite production of the PbHscB-Pbmei2-dKO clone, but only four data points are shown. Clarifying whether these points represent averages from multiple experiments or sporozoite counts from individual mosquitoes would be helpful.

We thank the Reviewer for pointing out the missing information in the Figure 3 legend. Each data point shown in the graph of Figure 3B represents the mean of salivary gland sporozoites number from 10 mosquitoes for one experiment, meaning that four independent experiments are represented in the graph in Fig 3B. See below the new figure legend:

**Fig 3: *Pb*Mei2-KO and *Pb*HscB-*Pb*Mei2-dKO do not affect oocyst formation and sporozoite production. (A)** Midguts of infected mosquitoes were dissected (n=10) and imaged on day 7 and day 14 post-feeding. Images were acquired using a 5x objective and analyzed using ImageJ software: mCherry signal was binarized using the threshold function and oocyst size was measured with the analyze particles function. The presented result is a representative of three experiments and shows 240 oocysts per parasite line as violin plot with individual oocyst sizes and medians with interquartile ranges. Kruskal-Wallis test did not result in a significant difference (ns) between WT and modified parasite line oocysts in neither at day 7 nor at day 14 post feeding. (p_day7 1868-dKO_=0.5731, all others >0.9999). **(B)** Infected salivary glands were dissected on indicated time points, homogenized and the number of sporozoites was determined with a Neubauer counting chamber. Each data point represents the mean of salivary gland sporozoites number from 10 mosquitoes determined in four independent experiments. The significance of the results was calculated using the One-way Anova test.

An additional experiment to demonstrate the immunogenicity of this double knockout parasite, such as vaccinating mice followed by a challenge with infectious sporozoites, could further validate the study, though protection is likely, given the late arrest of the parasite.

We appreciate the reviewer’s suggestion to include an additional challenge experiment to further validate the immunogenicity of the double knockout parasite. While we agree that such an experiment would be valuable, we would like to point out that this type of study is currently planned in our laboratory. However, given the nature of the planned experiments, which involve a range of parameters such as varying numbers of parasites, multiple time points, and the investigation of long-lasting immunity, the full completion of these experiments will take an estimated nine months.

We believe that these planned studies will greatly contribute to the understanding of the long-term immune response, but they are beyond the scope of the current manuscript. In this paper, we have provided robust data demonstrating a solid attenuation of the new double attenuated strain. As the reviewer kindly acknowledges, protection is expected due to the late arrest of the parasite, and we believe that the current results provide sufficient evidence for the conclusions drawn. We therefore respectfully suggest that this additional experiment, while important, be included in a future follow-up study to expand upon the present findings.

The discussion section, while informative, is somewhat lengthy and contains redundancies. The "experimental procedure" section also could benefit from clearer and more concise language, particularly regarding the redundancy between "Animal experiments conducted at the University of Bern" and "in vivo experiments."

We have removed some section in “Animal experiments conducted at the University of Bern” as they were indeed redundant with the “in vivo experiments” section. We rephrased or deleted parts that were redundant within the Discussion.

In conclusion, addressing these points will enhance the manuscript. Developing robustly genetically attenuated Plasmodium parasites to minimize breakthrough risks is crucial for advancing malaria vaccines. Blocking parasite development at a late liver stage to ensure broad antigenic presentation and optimize the immune response is a significant challenge. The strategy of targeting different pathways for attenuation, as demonstrated in this study, is a promising approach to prevent breakthrough infections.

We highly appreciate the encouragement of the Reviewer comment and really hope that we and others are paving the path to generate next generation of live attenuated parasites as future vaccines against malaria.
